# Choosing Wisely Philippines: ten low-value or harmful practices that should be avoided in cancer care

**DOI:** 10.3332/ecancer.2022.1424

**Published:** 2022-07-07

**Authors:** Frederic Ivan Ting, Crizel Denise Uy, Katrina Gaelic Bebero, Danielle Benedict Sacdalan, Honey Sarita Abarquez, Grace Nilo, Buenaventura Ramos Jr, Dennis L Sacdalan, Arnold John Uson

**Affiliations:** 1 Riverside Bacolod Cancer Care Center, Bacolod 6100, Philippines; 2 San Juan De Dios Hospital, Manila 1000, Philippines; 3 Bishop Joseph Regan Memorial Hospital, Davao Del Norte 8100, Philippines; 4 University of Toronto, Toronto, ON M5G 1N6, Canada; 5 Davao Doctors Hospital, Davao City 8000, Philippines; 6 St. Luke’s Medical Center – Global City, Manila 1000, Philippines; 7 Cebu Doctors University Hospital, Cebu City 6000, Philippines; 8 Philippine General Hospital, University of the Philippines, Manila 1000, Philippines; 9 Perpetual Succour Hospital, Cebu City 6000, Philippines

**Keywords:** Choosing Wisely, Philippines, cancer care, practices

## Abstract

The Choosing Wisely Philippines campaign is an initiative that identifies low-value or potentially harmful practices that are relevant to patients with cancer in the Philippines. The main purpose of these initiatives is to facilitate quality improvement systems and maximise patient outcomes. Of the ten practices identified, four are new recommendations, and six are modified adaptations from previous Choosing Wisely initiatives in the USA and Africa. Recommendations in the final list include interventions involving diagnosis (two practices), treatment (five practices), palliative and supportive care (two practices) and surveillance (1 practice).

Choosing Wisely Philippines (CWP) builds on work from the Choosing Wisely (CW) initiatives in the United States, Canada, India and Africa [1–4] and aims to identify low-value and unnecessary practices that result in inferior outcomes. These initiatives also intend to start the discussion between physicians, patients, their families and policymakers on ensuring high-quality cancer care while avoiding the use of unnecessary procedures and treatments. Furthermore, the identification of low-value or harmful practices can facilitate subsequent quality improvement initiatives and maximise patient outcomes.

The CW movement is driven by physicians who identify common medical practices that do not offer benefits to patients and may cause harm. CWP comes as the third CW campaign in low- and middle-income countries and the first in Southeast Asia. This Philippine Society of Medical Oncology (PSMO)-initiated campaign aims to facilitate a discussion among cancer-treating physicians including oncologists and non-oncologists, surgeons, paramedical staff in cancer care, patients and policymakers. The discussions will focus on efforts to reduce the use of low-value practices across the country, with an overall goal of providing the highest quality of care for patients with cancer.

The archipelagic nature of the Philippines makes it difficult for patients to travel to large academic centres that are centred in the major cities [5], and the low physician to patient ratio of cancer specialists in the more than 7,100 islands of the country makes cancer care delivery highly variable. Furthermore, suboptimal compliance to consensus-based guidelines is not uncommon in areas where the ideal diagnostic or therapeutic options could not be pursued due to a lack of available resources, manpower and/or logistical concerns. It is therefore important that professional and academic organisations such as the PSMO advocate for a unified way of practicing oncology wisely and for stewardship of resources as the Philippines moves towards universal health coverage and implement its national cancer control programmes [6].

In this article, we describe the methods used, and the results of, identifying a list of ten cancer care practices that are used in the Philippines, which are considered of low value, unnecessary or harmful to the patient.

## Consensus process

The Clinical Consensus Committee of the PSMO is the society’s arm in developing position statements that provide clinical guidance to its members based on careful examination of the best available scientific data. The nine committee members are situated in the three different island groups of the Philippines to ensure representation of the different regional contexts and practices. The scope of the initial list of the position statements considered included practices from cancer diagnosis, treatment and palliative and supportive care. The initial list was developed through a review of existing CW statements in the United States, Canada, Africa and India. New statements that apply to the practice of oncology in the Philippines were also provided by the PSMO through the suggestion of the committee members. The committee used six major guiding principles in identifying the final list and included: relevance and applicability in the Philippine setting; published evidence of low value/harm; clarity of statements; cost; measurability and frequent use in the Philippines. A voting threshold of 50% or more was used to include an individual recommendation on the short list. After a round of voting was completed, the committee further discussed each point on the short list (a yes or no decision was made by each committee member for each recommendation on the basis of its importance) and a consensus-based final list was created.

## Top Ten List

This list (Table 1) is meant to augment the existing Choosing Wisely USA, Canada and International lists with specific Philippine context. As such, the lack of inclusion of any existing published practices does not imply nonsupport of those practices for being low value or harmful.

1. **Do not initiate cancer treatment without confirming the diagnosis, defining the extent of the cancer and discussing the intent of treatment with the patient**.
  A diagnosis of cancer is life-changing news for any patient that receives it. It is therefore incumbent upon the attending physician to ensure that such a disclosure is rooted on a careful evaluation of all available clinical and diagnostic information. The extent and burden of disease will inform the appropriate treatment plan for the individual patient [4]. Whenever possible, this plan should be made within the context of a multidisciplinary team [3, 4, 8]. Treatment intent ultimately directs the treatment plan. Whether the therapeutic undertaking to be initiated will be curative or palliative needs to be clearly communicated to the patient as soon as the diagnosis has been established. Clarifying treatment goals from the outset will help to ensure that any care a patient will receive will best align with their values and preferences. It may be that a patient will decline a more aggressive course of care in favour of another more palliative approach if they come to understand the intent and potential toxicities associated with a planned treatment [4]. It is essential to respect the wishes of patients in treatment decision-making because they are ultimately subjected to the outcomes of these decisions [9].
2. **Do not use serum tumour markers indiscriminately for the screening and diagnosis of cancer.**
  Tumour markers are measurable biomolecules, typically proteins that are either made at higher amounts by cancer cells relative to normal cells or are produced by the body in response to a tumour. These substances can be isolated in the blood, urine or other tissues or bodily fluids [10, 11]. The ease of access to tumour markers makes these attractive as non-invasive surrogates to tumour tissue in the diagnosis of cancer. However, tumour markers suffer from several fundamental issues that make these ill-suited as stand-alone tests for the screening and diagnosis of cancer. Tumour markers can be highly non-specific, and their levels can vary widely across individuals with or without cancer.
  Both malignant and non-malignant processes, as well as medications can cause an increase in the levels of tumour markers. Conversely, early-stage cancers, which benefit most from screening, may not demonstrate elevated levels of tumour markers [11, 12]. The significant risk for false positive and false negative results argues strongly against their routine use. This is particularly important when framed within the context of the biopsychosocial implications of an incorrect diagnosis. However, once a particular tumour has been diagnosed, tumour markers may be used as a way of monitoring the success of treatment [11]. Thus, its use should be done with careful clinical judgment.
3. **Do not forget to discuss the value of biomarker testing for specific solid tumours where targeted treatments have proven benefits.**
  The availability of targeted treatment has revolutionised treatment decision-making in cancer in the last decade. Best exemplified in lung cancer, multiple biomarker-defined tumours [e.g. Programmed Death Ligand - 1 (PD-L1), Epidermal Growth Factor Receptor (EGFR), Anaplastic Lymphoma Kinase (ALK) mutations, among others] show strong evidence showing superior clinical outcomes (e.g. longer overall and progression free survival), while avoiding the toxicities of cytotoxic chemotherapy [13]. In settings where appropriate diagnostic tools and treatment are accessible, omitting biomarker testing can result in missed opportunities for patient benefit [14].
4. **Do not decide treatment of potentially curable cancers without inputs from a multidisciplinary oncology team.**
  Collaboration of relevant health care professionals through a multidisciplinary team has long been established as a cornerstone of cancer care worldwide [15]. The Multi-disciplinary Team approach facilitates best decisions about each patient’s diagnosis, stage, treatment and support, resulting to improved survival, adherence to guide-line based treatment and better quality of life [16]. Particularly for curable cancer, discussions on the applicability of neoadjuvant/adjuvant chemotherapy, timing of surgery and benefit of radiation should be made through thoughtful collaboration among subspecialities.
5. **Do not use surgery as the initial treatment without considering presurgical (neoadjuvant) systemic therapy and/or radiation for certain cancer types and stages where it is effective at improving local cancer control, quality of life or survival.**
  Numerous studies have shown that neoadjuvant treatments such as chemotherapy, hormonal therapy and/or radiation therapy followed by surgery have led to improved outcomes for patients with certain cancer types in the locally advanced stages such as breast, rectal, gastric, pancreatic, prostate and non-small cell lung cancer among others [17–21]. Presurgical treatment may decrease the size of the primary tumour, improves resectability, reduces local recurrence and may even improve the patients’ quality of life [22]. Unfortunately despite these data, a significant number of patients still undergo upfront surgery despite having indications for neoadjuvant treatment leading to suboptimal patients’ outcomes. The absence of a regular multidisciplinary team approach to cancer care makes this a common problem across the different regions of the country.
6. **Do not use combination cytotoxic chemotherapy when treating an individual for metastatic breast cancer unless the patient needs a rapid response to relieve tumour-related symptoms; instead, use a single cytotoxic agent.**
  Some studies show that combination chemotherapy (use of multiple cytotoxic drugs) for metastatic breast cancer may control tumour growth for a longer time than when treating with a single agent [23]. However, the use of combination chemotherapy has not been shown to increase the patients’ overall survival. Although combination chemotherapy may be useful in situations where the cancer tumour burden must be reduced quickly because it is causing significant symptoms, is life threatening or causing visceral crises, its use must be balanced with its more frequent side effects [24, 25]. Use of non-cytotoxic treatments such as hormonal/endocrine therapies and/or targeted therapies should be considered for patients with hormone receptor and/or Human Epidermal Growth Factor Receptor-2-positive metastatic breast cancer [24–26].
7. **Do not use cancer-directed therapy for patients with solid tumours with ALL of the following characteristics: low PS (3 or 4), no benefit from prior evidence-based interventions and no strong evidence supporting the clinical value of further anti-cancer treatment. Instead, focus on symptom relief and palliative care.**
  Several studies show that patients with poor Eastern Cooperative Oncology Group Performance Status (PS) (3–4) do not benefit from systemic therapy and may suffer from treatment-related toxicity leading to poor quality of life. [27–31] For these patients, the focus of care should be on the management of their symptoms and the provision of palliative care. Exceptions to this principle include patients where the malignancy itself is causing the poor ECOG PS and/or malignancies that are highly sensitive to chemotherapy and have high chances of cure even in advanced stages such as germ cell tumour, lymphoma and testicular cancer among others.
8. **Do not use whole body PET-CT scans to detect recurrence after completing curative treatment for asymptomatic patients with early-stage solid tumours.**
  The use of PET-CT scan is not superior over the recommended standard modalities in the surveillance of most solid tumours after a curative intent primary treatment. This can be a potential source of anxiety, unnecessary exposure to radiation and undue expenses and has not been proven to increase survival nor quality of life when used in this setting [25,32–35]. PET-CT scan should be limited to diseases wherein the detection of recurrence is impossible with the other imaging techniques. Do not order the test to detect recurrent cancer in asymptomatic patients if there is no realistic expectation that early detection of recurrence can improve survival or quality of life [4].
9. **Avoid the use of Granulocyte - Colony Stimulating Factor (G-CSF) for primary prevention of febrile neutropenia for patients with less than 10% risk for this complication.**
  Febrile neutropenia is a dreaded treatment-related complication in cancer; however, the use of G-CSF as primary prophylaxis is limited to patients with at least 20% risk. Careful consideration of other factors such as age and co-existing comorbidities should be observed in patients with intermediate risk (10%–20%). Patients with prior neutropenia may receive secondary prophylaxis if delay in treatment or dose reduction can cause detrimental outcomes [36–38]. During the COVID-19 pandemic, G-CSF prophylaxis indication is expanded for both high risk (>20%) and intermediate risk, specifically for elderly with comorbidities [39].
10. **Do not forget to discuss herbal medications including its potential harmful consequences while on active cancer treatment.**
  There is no herbal supplement approved for the treatment of cancer by the Philippine Food and Drug Administration and other national or international agencies. Herbal medications also have inherent risks of toxicity when used alone and have various drug interaction potential when taken together with standard medical treatment. Thorough careful discussion and proper patient education on this matter should be exercised at every opportunity by the clinicians [40–43].

## Discussion

The PSMO initiated the CWP project to identify ten practices that can be unnecessary or potentially harmful to patients with cancer. The relevance of CWP is shown by the broad spectrum of its reach where the entire cancer continuum is involved from diagnosis, treatment (curative and palliative) and surveillance.

This work was guided by previous CW initiatives (USA, Canada, India and Africa), where the PSMO Clinical Consensus Committee primarily sought to create a list that would provide specific Philippine context to its recommendations. As such, the absence of any Choosing Wisely USA, Canada, India or Africa does not mean disagreement about those practices having low value or harmful; it simply shows that more common practices that are particularly relevant to the Philippine context exist.

The CW Philippines list does not intend to replace discussions between physicians and patients or independent clinical judgment. The list was crafted to promote a patient-centred approach by shared decision-making. Furthermore, it is possible that new evidence might emerge in the future, and regular review of these practices and supporting evidence is a must.

Creating the CWP is only the first step in efforts to decrease the delivery of low-value care in the Philippines, which will hopefully reduce the financial burden associated with cancer care. To further strengthen its reach, the PSMO in partnership with ecancer will be crafting scientific modules of each of the key recommendations. Outputs of these modules will be presented through short video clips which is easily understandable and shared across multiple media platforms. Furthermore, the PSMO and ecancer will be holding a 2-day summit in the Philippines where multiple oncology practitioners and patient groups around the country will be invited to discuss the CWP key recommendations together with other topics of interest. The CWP is likewise in alignment to the WHO’s call for ‘more health for the money’ that encourages health systems to choose resources wisely where ten leading sources of inefficiencies were identified including the inappropriate use of medications and the overuse of investigations and procedures [44].

The strength of this work stems on the solid evidence that serves as the foundation of each key recommendation. Furthermore, the CWP list can be improved in the future by collaborating with other oncologic subspecialities together with patient groups.

## Conclusion

The PSMO, through its Clinical Consensus Committee, has spearheaded the CWP campaign that identified ten low-value and potentially harmful practices in cancer care. These recommendations are intended to promote discussions among patients and physicians, and encourage shared decision-making. Creating this list is only the first step of this initiative, and the ultimate goal is to improve the quality of cancer care that we provide patients in the Philippines thereby also improving survival and quality of life.

## Funding

None of the authors received any form of financial reward in the creation of this article.

## Conflicts of interest

All authors have no conflicts of interest to declare.

## Figures and Tables

**Table 1.** CWP final list of low-value or harmful practices that should be avoided in cancer care.

	Origin of recommendation	Revisions made to original recommendation
Do not initiate cancer treatment without confirming the diagnosis, defining the extent of the cancer and discussing the intent of treatment with the patient.	Choosing Wisely Africa [4]	Yes
Do not use serum tumour markers indiscriminately for the screening and diagnosis of cancer.	New suggestion	Not applicable
Do not forget to discuss the value of biomarker testing for specific solid tumours where targeted treatments have proven benefits.	New suggestion	Not applicable
Do not decide treatment of potentially curable cancers without inputs from a multidisciplinary oncology team.	Choosing Wisely USA [1, 7]	No
Do not use surgery as the initial treatment without considering presurgical (neoadjuvant) systemic therapy and/or radiation for certain cancer types and stages where it is effective at improving local cancer control, quality of life or survival.	Choosing Wisely Africa [4]	No
Do not use combination cytotoxic chemotherapy when treating an individual for metastatic breast cancer unless the patient needs a rapid response to relieve tumour-related symptoms; instead, use a single cytotoxic agent.	Choosing Wisely USA [1]	Yes
Do not use cancer-directed therapy for patients with solid tumours with ALL of the following characteristics: low performance status (PS) (3 or 4), no benefit from prior evidence-based interventions and no strong evidence supporting the clinical value of further anti-cancer treatment. Instead, focus on symptom relief and palliative care.	Choosing Wisely USA and Africa [1, 4]	Yes
Do not use whole body Positron Emission Tomography - Computed Tomography (PET-CT) scans to detect recurrence after completing curative treatment for asymptomatic patients with early-stage solid tumours.	New suggestion	Not applicable
Avoid the use of granulocyte-colony stimulating factor (G-CSF) for primary prevention of febrile neutropenia for patients with less than 10%–20% risk for this complication.	Choosing Wisely USA [1]	Yes
Do not forget to discuss about alternative/herbal medications including its potential harmful consequences while on active cancer treatment.	New suggestion	Not applicable
